# Survey data of rearing practices applied throughout the life of beef heifers from 45 mountain farms in France and main parameters of the related carcasses

**DOI:** 10.1016/j.dib.2022.107850

**Published:** 2022-01-20

**Authors:** Valérie Monteils, Cécile Sibra

**Affiliations:** Université Clermont Auvergne, INRAE, VetAgro Sup, UMR Herbivores, 63122 St-Genès-Champanelle, France

**Keywords:** Rearing practices, Whole life, Beef heifers management, survey questionnaire, Carcass characterisation

## Abstract

This paper presents data of rearing practices collected by survey from 45 beef heifers breeders. All the breeders were members of ‘Génisse Fleur d'Aubrac’ protected geographical indication (PGI). The surveys were conducted face-to-face using a questionnaire which addressed the rearing practices applied throughout the animals’ life. The animals’ life was considered in 3 periods: suckling, growth and fattening periods. The rearing practices (n=105) addressed a wide range such as the dietary compositions in stall and on pasture, the duration of each life period and the age of heifers at each change, the prophylactic treatments, the birth and slaughter dates. Both quantitative and qualitative variables were used to characterise the management system. The parameters (n=7) of the carcasses produced (n=636) in these farms were collected from the only slaughterhouse of the PGI.

## Specifications Table

**Table:** 

Subject	Animal Science
Specific subject area	Animal breeding
Type of data	Tables (Excel)
How data were acquired	Breeders surveys based on a survey questionnaire and collection of carcass data from slaughterhouse
Data format	Raw
Parameters for data collection	The survey included only breeders members of the ‘Génisse Fleur d'Aubrac’ protected geographical indication (PGI). The carcass data were collected from the only slaughterhouse authorised in the PGI specification.
Description of data collection	Data were collected in 2015 using face-to-face survey with 45 breeders for the rearing practices applied throughout the animals’ life. The original survey questionnaire in French and the translated version in English are provided in Supplementary files. The carcass data of the heifers slaughtered in 2014 were collected directly from the slaughterhouse.
Data source location	Aubrac, Massif central, France
Data accessibility	With the article
Related research article	[1] V. Monteils, C. Sibra, Rearing practices in each life period of beef heifers can be used to influence the carcass characteristics, Ital. J. Anim. Sci. 18 (2019) 734–745. https://doi.org/10.1080/1828051X.2019.1569486. [2] V. Monteils, C. Sibra, Identification of combinations of influential rearing practices applied during the heifers’ whole life on the carcass quality by the decision tree method, Livest. Sci. 230 (2019) 103823. https://doi.org/10.1016/j.livsci.2019.103823. [3] V. Monteils, C. Sibra, C. Laurent, Determination of rearing practices combinations increasing the carcase weight according to the heifers slaughter age by the decision tree method, Ital. J. Anim. Sci. 20 (2021) 1851–1862. https://doi.org/10.1080/1828051X.2021.1988738.

## Value of the Data

• The data presents the rearing practices applied along the whole life of beef heifers.
• The data can be used to characterise the management system of beef heifers in the studied area and to compare it with other regions and other productions.
• The data can be used to characterise the carcass properties of animals produced according to the specification of the PGI ‘Génisse Fleur d'Aubrac’.
• All the results can be used to relate the rearing management and the carcass properties.

## 1 Data Description

The dataset is presented in a single file with 4 sheets:

- the first sheet presents the definition of the 123 variables used in the survey questionnaire.
- the second sheet presents the general information of the 45 farms (11 variables). Each line represents a farm.
- the third sheet presents the raw data of rearing practices applied throughout the life of animals (105 variables). Each line represents a group of animals within a farm. Livestock management is described to characterise the animal group (7 variables, columns C to I), suckling period, from birth to weaning (32 variables, columns J to AO), growing period, from weaning to begin of fattening (33 variables, columns AP to BV) and fattening period, from begin of fattening to slaughter (33 variables, columns BW to DC).
- the fourth sheet presents the raw data of the 636 carcasses collected in slaughterhouse (7 variables). Each line corresponds to an animal with the correspondence of the group and the farm of origin.

The missing data, i.e. data that could not be collected in the farms, are mentioned “MD”. The purposeless data, for example data related to rearing practices in stall for a group conducted on pasture only, are mentioned “NApp” for “not applicable”.

## 2 Experimental Design, Materials and Methods

**Fig. 1:**
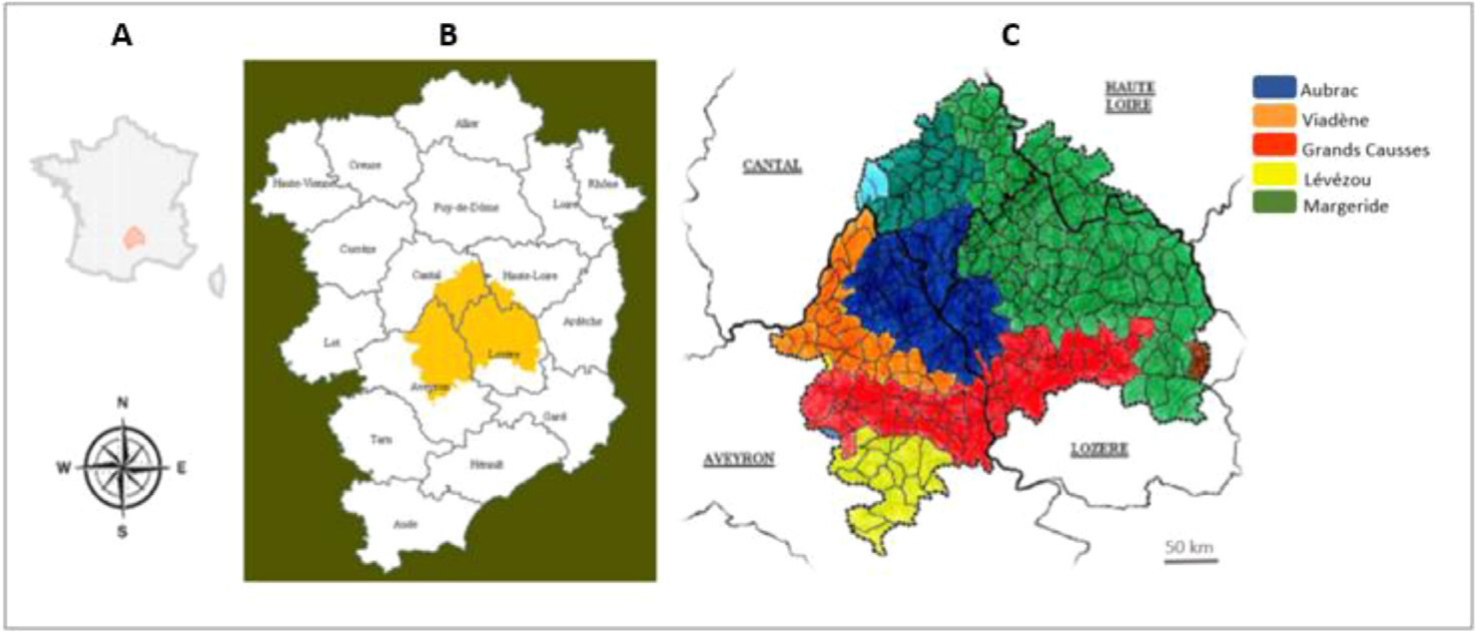
Location of the ‘Génisse Fleur d'Aubrac’ PGI area in France (A, [5]) and in the Massif central (B), and location of the 5 natural areas where the surveys were carried out (C, [6]).

The ‘Génisse Fleur d'Aubrac’ PGI had 190 breeders members. The breeders who participated in this study had to be breeders/fatteners, and to produce a significant number of labelled heifers (at least 15 in 2012-2013 and more than 5 in 2014) according to the PGI specifications [4]. Within this sub-population 45 breeders have agreed to take part to the study, i.e. almost 24% of the number. The production area is located on the Aubrac plateau, in a grassland mountain area in the Massif Central in France (Fig. 1). The main guidelines of the specification concern the breed of the heifers (cross-bred Aubrac x Charolais), their management (extensive system with a significant amount of grazing and no maize during fattening) and the slaughter age (between 24 and 42 mo). As the proportion of farms taking part to the study and their distribution throughout the production area, the sample can be considered representative of the total population.

The rearing practices were collected through a single face-to-face survey in 2015 with the breeder in charge of rearing of the heifers under the label ‘Génisse Fleur d'Aubrac’ PGI. The survey questionnaire addressed some general information about the farm and the rearing practices applied to animals throughout their lives. The practices were characterised mainly by the stall and pasture feeding practices (nature and quantity), the duration of each diet distribution and their correspondence to the dates. The birth and slaughter dates, the suckling method and the prophylactic treatments (vaccination, antiparasitics) were also gathered. The rearing practices were collected per group of animals, i.e. animals physically together and receiving the same rearing practices. A specific diagram was used to report the batch management, to identify all groups of heifers in a farm and to follow them throughout the animals’ life [7]. All information related to the management of animals’ groups was reported at the corresponding dates: such as stalling or pasture, weaning, fattening start, vaccination and antiparasitic treatments, arrival and exit of animals from the group, and all changes in diet which were also detailed in tables (forages and concentrates natures and quantities).

The survey questionnaires (the original version in French and the translated version in English) including the batch management diagram with the tables for diet compositions are available in Supplementary material. An example of a batch management diagram completed during a survey is also presented in Supplementary material.

The data from the 636 carcass produced in 2014 by the 45 breeders were collected at the individual level from the single slaughterhouse authorised in the PGI specification (Slaughterhouse of Gévaudan in Marvejols, France). All carcasses were weighed 1 hour after slaughter and characterised (conformation and fat score) according to the EUROP system [8].

## Ethics Statement

All data collected in farms and in the slaughterhouse were anonymised after the data collection. The surveys were done with voluntary breeders and carcass data were collected from a slaughterhouse which respected the French legislation in force at the time of the study. The device used did not change the conditions under which the animals were raised and slaughtered. No ethical approval was required.

## CRediT authorship contribution statement

**Valérie Monteils:** Supervision, Conceptualization, Methodology, Data curation, Writing – original draft, Writing – review & editing, Validation. **Cécile Sibra:** Conceptualization, Methodology, Data curation, Writing – original draft, Writing – review & editing, Visualization.

## Declaration of Competing Interest

The authors declare that they have no known competing financial interests or personal relationships which have or could be perceived to have influenced the work reported in this article.

## Appendix Supplementary Materials

Image, application 1

Image, application 2

Image, application 3

Image, application 4
