# Supplementary material for: Survey data of rearing practices applied throughout the life of beef heifers from 45 mountain farms in France and main parameters of the related carcasses
Source: Data Brief. 2022 Jan 20;41:107850. doi: 10.1016/j.dib.2022.107850 (PMC8802835; doi:10.1016/j.dib.2022.107850)
Supplement: Supplementary file 1 [file mmc1.docx]

**SURVEY QUESTIONNAIRE**

**In blue are the instructions for the interviewer's use**

**Interviewers name: Date:**

**Farm name: Respondent name:**

**Full adress :**

**PART I: GENERAL DESCRIPTION OF THE FARM**

Quick history of the farm:

Farmstead elevation (m):

In which natural area is the farm located?

Aubrac / Ségala / Lévézou / Vallon de Marcillac / Viadène / Grands Causses / Cévennes / Margeride

How much is the human work force (HWU)?

Do you have other suckling cattle productions? Yes/ No

Do you have other productions than suckling cattle? Yes/ No

Number of calvings per year:

UAA of the farm (ha):

Detail of surface area:

Grass area (ha):

Forage maize area (ha):

Cereal area (ha):

Frequency of forage purchase: Never / every year / every 2 years / if hazards

**PART II: MANAGEMENT OF THE PGI HEIFERS ‘GENISSES FLEUR D’AUBRAC’**

In this section, the record of the heifers’ management will only concern the animals slaughtered in 2014. The aim is to go back in time to trace the life of the animal and the rearing practices that were applied.

What is the suckling method?

Accompanied (the farmer led the calf to her mother twice a day for suckling) / Free (the calf stayed always with her mother and suckled ad libitum) / Both (the both methods)

In how many animals’ groups are the heifers managed? (a group is defined such as animals physically together and receiving the same rearing practices)

What is the composition of the different groups:

| Animals’ group ID | Group 1 | Group 2 | Group 3 | Group 4 | Group 5 | Group 6 | Group 7 | … |
| --- | --- | --- | --- | --- | --- | --- | --- | --- |
| Number of heifers in the group |  |  |  |  |  |  |  |  |
| Identification of the heifers in the group (heifers ID) |  |  |  |  |  |  |  |  |

For the rest of the questionnaire, we will reason for the different groups separately.

The following batch management diagram will allow us to trace the life of the animals and to provide information on the rearing practices applied as well as the major events concerning: feeding, housing, treatments, breeding, etc.

**Batch management diagram**

**Principe:**

All groups identified in 2014 must be reported on the batch management diagram, indicating the Group ID assigned in the previous question.

A group must appear in a column with the identification of events throughout the life of the animals in it.

**For each group:**

Using the legend and specifying the dates, place on the diagram:

- the periods of birth, weaning, slaughter
- the start of breeding of heifers
- changes in location (putting out to grass and putting into stall) and types of housing (free stall or stanchion barn)
- prophylactic events carried out and types of treatment
- group changes, arrivals and exits of animals from groups to monitor numbers

Diet changes should also be indicated on the diagram by identifying the period of diet distribution (start and end dates) and by indicating numbers (different diet numbers as soon as there is a difference in composition (nature or quantity of feedstuffs). Diets for the mother/calf pair will be noted MC_diet number; post-weaning rations for heifers will be noted H_diet number.

All diet numbers should be recorded in the Table of supplied diets to give details of each diet in terms of nature and quantity of feedstuffs.

Different groups may be fed the same diet at the same time, so the quantities distributed to the different groups and the numbers in each group should be clearly identified.

Pasture will be considered as a full-fledged diet and any supplements provided must be identified. The fattening period must be explicitly identified, whether it is carried out in stall or on pasture, with clear identification of the start and end dates and the composition of the diet. Dietary transitions should also be identified by a specific diet number.

**Legend of the batch management diagram**

**Animals type**

Mother/calf pair : in red

Heifer : in bleu

**Animal location**

Pasture

Stanchion barn

Free stall

in full or alternating day and night

in full or alternating day and night

**Events in the animal life**

Spreading of slaughter over time

Start and end dates of a group :

Diet number : **MC_n ou H_n**

W

Weaning period :

Fattening period :

F

Dehorning :

D

Start and end dates of births period : (report the calving number between the two arrows)

Animal exit (x for the number) :

Animal arrival (x for the number) : specify purchase/sale/group change

Breeding with a bull (
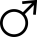
 ) or with artificial insemination using frozen semen (AI).

Prophylactic treatments: in green, specify the intervention type and the animals concerned. Death:
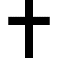
 (specify the number)

**Batch management diagram**

| 2010 | Jun |  |  |  |  |
| --- | --- | --- | --- | --- | --- |
|  | Jul |  |  |  |  |
|  | Aug |  |  |  |  |
|  | Sep |  |  |  |  |
|  | Oct |  |  |  |  |
|  | Nov |  |  |  |  |
|  | Dec |  |  |  |  |
| 2011 | Jan |  |  |  |  |
|  | Feb |  |  |  |  |
|  | Mar |  |  |  |  |
|  | Apr |  |  |  |  |
|  | May |  |  |  |  |
|  | Jun |  |  |  |  |
|  | Jul |  |  |  |  |
|  | Aug |  |  |  |  |
|  | Sep |  |  |  |  |
|  | Oct |  |  |  |  |
|  | Nov |  |  |  |  |
|  | Dec |  |  |  |  |
| 2012 | Jan |  |  |  |  |
|  | Feb |  |  |  |  |
|  | Mar |  |  |  |  |
|  | Apr |  |  |  |  |
|  | May |  |  |  |  |
|  | Jun |  |  |  |  |
|  | Jul |  |  |  |  |
|  | Aug |  |  |  |  |
|  | Sep |  |  |  |  |
|  | Oct |  |  |  |  |
|  | Nov |  |  |  |  |
|  | Dec |  |  |  |  |

**Batch management diagram (continued)**

| 2013 | Jan |  |  |  |  |
| --- | --- | --- | --- | --- | --- |
|  | Feb |  |  |  |  |
|  | Mar |  |  |  |  |
|  | Apr |  |  |  |  |
|  | May |  |  |  |  |
|  | Jun |  |  |  |  |
|  | Jul |  |  |  |  |
|  | Aug |  |  |  |  |
|  | Sep |  |  |  |  |
|  | Oct |  |  |  |  |
|  | Nov |  |  |  |  |
|  | Dec |  |  |  |  |
| 2014 | Jan |  |  |  |  |
|  | Feb |  |  |  |  |
|  | Mar |  |  |  |  |
|  | Apr |  |  |  |  |
|  | May |  |  |  |  |
|  | Jun |  |  |  |  |
|  | Jul |  |  |  |  |
|  | Aug |  |  |  |  |
|  | Sep |  |  |  |  |
|  | Oct |  |  |  |  |
|  | Nov |  |  |  |  |
|  | Dec |  |  |  |  |

**Table of supplied diets**

| Diet number | Group number and size | **For the group**  Nature and quantity of supplied forages  Specify the unit:  Dry matter or raw matter  /day or /meal  For pasture: grass or forage supplement | **For the group**  Nature and quantity of supplied concentrates  Specify the unit:  Dry matter or raw matter  /day or /meal  For pasture: concentrate supplement | Distribution number /day | | Distribution method (mixed forages/concentrates, mixed *concentrates, ad* *libitum*, haystack, open silo) |
| --- | --- | --- | --- | --- | --- | --- |
|  |  |  |  | Forage | Concentrate |  |
|  |  |  |  |  |  |  |
|  |  |  |  |  |  |  |
|  |  |  |  |  |  |  |
|  |  |  |  |  |  |  |
