# Supplementary material for: Survey data of rearing practices applied throughout the life of beef heifers from 45 mountain farms in France and main parameters of the related carcasses
Source: Data Brief. 2022 Jan 20;41:107850. doi: 10.1016/j.dib.2022.107850 (PMC8802835; doi:10.1016/j.dib.2022.107850)
Supplement: Supplementary file 2 [file mmc2.docx]

**QUESTIONNAIRE D’ENQUETE**

**En bleu les instructions destinées aux enquêteurs**

**Nom des enquêteurs : Date :**

**Nom de l’exploitation : Nom de l’enquêté :**

**Adresse complète :**

**PARTIE I : DESCRIPTION GENERALE DE L’EXPLOITATION**

Historique rapide de l’exploitation :

Altitude du siège (m) :

Petite Région Agricole ou région naturelle :

Aubrac / Ségala / Lévézou / Vallon de Marcillac / Viadène / Grands Causses / Cévennes / Margeride

Nombre d’UTH :

Avez-vous d’autres ateliers bovins allaitants ? Oui / Non

Avez-vous d’autres ateliers que bovins allaitants ? Oui / Non

Nombre de vêlages par an :

SAU de l’exploitation (en ha) :

Détail des surfaces :

Surface en herbe (ha) :

Surface en maïs fourrage (ha) :

Surface en céréales (ha) :

Fréquence d’achat de fourrage : Jamais / Tous les ans / Tous les deux ans / En cas d’aléas

**PARTIE II : PRODUCTION DES GENISSES FLEUR D’AUBRAC**

Dans cette partie, le relevé de la conduite mise en œuvre portera uniquement sur les génisses Fleur d’Aubrac abattues en 2014. Le but est de remonter dans le temps pour retracer la vie de l’animal et les pratiques d’élevage qui ont été appliquées.

Quel système de tétée est utilisé ?

Accompagné (l’éleveur conduit le veau à sa mère 2 fois par jour pour téter) / Libre (le veau est toujours avec sa mère et tète à volonté) / Les deux (les 2 méthodes sont utilisées)

Les génisses sont conduites en combien de lots ? *(Un lot est défini comme un groupe d’animaux physiquement ensemble et recevant les mêmes pratiques d’élevage)*

Quelles est la composition des différents lots ?

| Identifiant du lot | Lot 1 | Lot 2 | Lot 3 | Lot 4 | Lot 5 | Lot 6 | Lot 7 | … |
| --- | --- | --- | --- | --- | --- | --- | --- | --- |
| Nombre de génisses dans le lot |  |  |  |  |  |  |  |  |
| Identification des génisses dans le lot |  |  |  |  |  |  |  |  |

Pour la suite du questionnaire, nous allons raisonner pour les différents lots de façon distincte.

Le schéma d’allotement suivant va permettre de retracer la vie des animaux et de renseigner les pratiques mises en œuvre ainsi que les évènements majeurs concernant : l’alimentation, le logement, les traitements, la mise à la reproduction…

**Schéma d’allotement**

**Principe**

Tous les lots identifiés en 2014 doivent être reportés sur le schéma d’allotement, en précisant le numéro attribué à la question précédente

Un lot doit apparaitre en colonne avec l’identification des évènements tout au long de la vie des animaux qui le composent

**Pour chaque lot :**

A l’aide de la légende et en précisant à chaque fois les dates, placez sur le schéma :

- Les périodes de naissance, de sevrage et d’abattage
- La mise à la reproduction des génisses

Les changements de localisation (mises à l’herbe et rentrées en bâtiment) et les types de logement (stabulation libre ou entravée, type de sol)

- Les évènements de prophylaxie réalisés et les types de traitements
- Les changements de lots, les entrées et les sorties d’animaux des lots pour suivre les effectifs

Faire apparaitre également les changements de ration en identifiant la période de distribution de la ration (dates de début et fin) et en indiquant des numéros (numéros de ration différents dès qu’il y a une différence dans la composition (nature des aliments ou quantité). Les rations concernant le couple mère/veau seront notées MV + n° ; les rations après sevrage concernant les génisses Fleur d’Aubrac seront notées G + n°.

Tous les identifiants de ration seront reportés dans le tableau d’alimentation pour avoir le détail de chaque ration en termes de natures et de quantités d’aliments.

Différents lots peuvent recevoir la même ration à un même moment, il faudra alors identifier clairement les quantités distribuées aux différents lots, et les effectifs de chaque lot.

Le pâturage sera considéré comme un type de ration à part entière, il faudra identifier les éventuels compléments apportés. La période de finition devra apparaitre explicitement, qu’elle soit réalisée en bâtiment ou au pâturage, avec une identification précise des dates de début et fin ainsi que la composition de la ration. Les transitions alimentaires devront également être identifiées par un numéro de ration propre.

**Légende du schéma d’allotement**

**Type d’animaux**

Couple mère/veau : **ROUGE**

Génisse Fleur d’Aubrac : **BLEU**

**Localisation des animaux**

Pâturage

Stabulation

Entravée

Stabulation libre

En totalité ou alternance jour/nuit

En totalité ou alternance jour/nuit

**Evènements dans la vie des animaux**

Etalement des abattages dans le temps

Début et fin d’un lot :

Ration n : **MVn ou Gn**

**S**

Période sevrage :

**F**

Période de finition

**E**

Ecornage :

Début et fin des périodes de naissance : (noter le nombre de vêlages entre les 2 flèches)

Sortie d’animaux :

Entrée d’animaux : préciser achat/vente/réallotement

Reproduction avec un taureau :
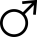
 ou par insémination artificielle (semence surgelée) : **IA**

Prophylaxie : VERT (noter le type d’intervention et les animaux concernés)

Mortalité :
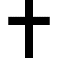
 (préciser le nombre)

**Schéma d’allotement**

| 2010 | Juin |  |  |  |  |
| --- | --- | --- | --- | --- | --- |
|  | Juil |  |  |  |  |
|  | Août |  |  |  |  |
|  | Sep |  |  |  |  |
|  | Oct |  |  |  |  |
|  | Nov |  |  |  |  |
|  | Dec |  |  |  |  |
| 2011 | Jan |  |  |  |  |
|  | Fev |  |  |  |  |
|  | Mar |  |  |  |  |
|  | Avr |  |  |  |  |
|  | Mai |  |  |  |  |
|  | Juin |  |  |  |  |
|  | Juil |  |  |  |  |
|  | Août |  |  |  |  |
|  | Sep |  |  |  |  |
|  | Oct |  |  |  |  |
|  | Nov |  |  |  |  |
|  | Dec |  |  |  |  |
| 2012 | Jan |  |  |  |  |
|  | Fev |  |  |  |  |
|  | Mar |  |  |  |  |
|  | Avr |  |  |  |  |
|  | Mai |  |  |  |  |
|  | Juin |  |  |  |  |
|  | Juil |  |  |  |  |
|  | Août |  |  |  |  |
|  | Sep |  |  |  |  |
|  | Oct |  |  |  |  |
|  | Nov |  |  |  |  |
|  | Dec |  |  |  |  |

**Schéma d’allotement (suite)**

| 2013 | Jan |  |  |  |  |
| --- | --- | --- | --- | --- | --- |
|  | Fev |  |  |  |  |
|  | Mar |  |  |  |  |
|  | Avr |  |  |  |  |
|  | Mai |  |  |  |  |
|  | Juin |  |  |  |  |
|  | Juil |  |  |  |  |
|  | Août |  |  |  |  |
|  | Sep |  |  |  |  |
|  | Oct |  |  |  |  |
|  | Nov |  |  |  |  |
|  | Dec |  |  |  |  |
| 2014 | Jan |  |  |  |  |
|  | Fev |  |  |  |  |
|  | Mar |  |  |  |  |
|  | Avr |  |  |  |  |
|  | Mai |  |  |  |  |
|  | Juin |  |  |  |  |
|  | Juil |  |  |  |  |
|  | Août |  |  |  |  |
|  | Sep |  |  |  |  |
|  | Oct |  |  |  |  |
|  | Nov |  |  |  |  |
|  | Dec |  |  |  |  |

**Tableau des rations distribuées**

| N° ration | Taille et N° du lot | **Pour le lot**  Nature et quantité de fourrages distribués :  Préciser l’unité : matière sèche (MS) ou matière brute (MB),  /jour ou /repas  Pour pâturage : mettre herbe, affouragement | **Pour le lot**  Nature et quantité de concentrés distribués :  Préciser l’unité : matière sèche (MS) ou matière brute (MB),  /jour ou /repas  Pour pâturage : complémentation | Nb de distribution /jour | | Mode de distribution (mélange fourrage/concentrés, mélange des concentrés, à volonté (botte foin ou ouverture silo)…) |
| --- | --- | --- | --- | --- | --- | --- |
|  |  |  |  | Fourrage | Concentré |  |
|  |  |  |  |  |  |  |
|  |  |  |  |  |  |  |
|  |  |  |  |  |  |  |
|  |  |  |  |  |  |  |
