# Supplementary figures and images for: Survey data of rearing practices applied throughout the life of beef heifers from 45 mountain farms in France and main parameters of the related carcasses
Source: Data Brief. 2022 Jan 20;41:107850. doi: 10.1016/j.dib.2022.107850 (PMC8802835; doi:10.1016/j.dib.2022.107850)

Example of a batch management diagram completed with a breeder during a survey


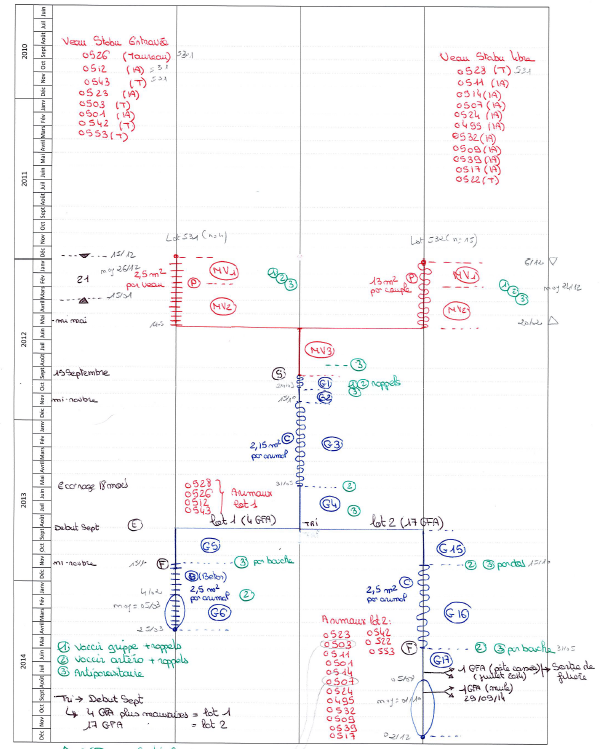

Supplement: Supplementary file 3 [file mmc3.docx]
